# Paper-Based Sensor Chip for Heavy Metal Ion Detection by SWSV

**DOI:** 10.3390/mi9040150

**Published:** 2018-03-27

**Authors:** Xiaoqing Wang, Jizhou Sun, Jianhua Tong, Xin Guan, Chao Bian, Shanhong Xia

**Affiliations:** 1 State Key Laboratory of Transducer Technology, Institute of Electrics, Chinese Academy of Sciences, Beijing 100190, China; wangxiaoqing15@mails.ucas.edu.cn (X.W.); jhtong@mail.ie.ac.cn (J.T.); hans9633@sina.com (X.G.); shxia@mail.ie.ac.cn (S.X.); 2 University of Chinese Academy of Sciences, Beijing 100190, China

**Keywords:** paper-based sensor, magnetron sputtering, SWSV, heavy metal ions

## Abstract

Heavy metal ion pollution problems have had a terrible influence on human health and the environment. Therefore, the monitoring of heavy metal ions is of great practical significance. In this paper, an electrochemical three-electrode system was fabricated and integrated on nitrocellulose membrane (NC) by the use of magnetron sputtering technology, which exhibited a uniform arrangement of porous structure without further film modification. This paper-based sensor chip was used for Cu^2+^ detection by square-wave stripping voltammetry (SWSV). Within the ranges of 5–200 μg·L^−1^ and 200–1000 μg·L^−1^, it showed good linearity of 99.58% and 98.87%, respectively. The limit of detection was 2 μg·L^−1^. On the basis of satisfying the detection requirements (10 μg·L^−1^), the integrated sensor was small in size and inexpensive in cost. Zn^2+^, Cd^2+^, Pb^2+^ and Bi^3+^ were also detected by this paper-based sensor chip with good linearity.

## 1. Introduction

Some of the heavy metal ions are necessary trace elements for human life activities, but they are mostly harmful to the environment and human health if their concentrations exceed the standard limit [1,2]. It is therefore important to be able to detect the concentration of heavy metal ions.

Metal ion detection methods [3] mainly include atomic spectroscopy [4], UV–visible spectrophotometry [5], mass spectrometry [6], electrochemical analysis [7], etc. Electrochemical analysis is recognized as a fast and sensitive method. As one of the electrochemical analysis methods, anodic stripping voltammetry is high in accuracy, widely applicable, and easy to miniaturize. The electrochemical analysis method relies on a three-electrode system (WE, working electrode; CE, counter electrode; RE, reference electrode) in which the working electrode is the core part and its performance is critical to the detection result. The working electrode is mostly a solid-state electrode. Usually, the sensitivity and specificity can be improved by sensing membrane modification [8,9,10,11]. Xing [12] et al. designed the N-type-graphene-modified electrode to achieve the goal of analyzing Ca^2+^, Pb^2+^, and Cu^2+^ simultaneously with high sensitivity. Wang [13] et al. used gold nanoparticles to modify a microelectrode chip, which resulted in a lower detection limit for detecting Pb^2+^ and Cu^2+^.

Since 2007, the advantages of paper-based materials have attracted more attention. They can be wildly used in public health, clinical diagnostics [14], paper microfluidics [15], environmental monitoring, etc. For example, Ainla [16] et al. designed electrically activated fluidic valves fabricated using textiles. The valves had fast response and low energy consumption. They can be used in paper-based microfluidic devices. Many researchers have also tried to prepare paper-based sensors for heavy metal ion detection [17,18,19]. Ensafi [20] et al. prepared low-cost screen-printed electrodes (SPE) and developed a signal enhancement method through gold nanoparticles. It had low limit detection for As^3+^. Feng [21] et al. designed hydrophobic areas on paper-based materials and sensor arrays were formed based on the structure of paper. According to the colorimetric method, it qualitatively distinguished Cu^2+^, Zn^2+^, Ni^2+^, Co^2+^, and some other heavy metal ions.

There are several kinds of methods for fabricating paper-based sensors, such as wax printing technology [22], inkjet printing technology [23], screen printing technology [24], etc. These methods are easy to operate but most of them are thick-film technologies. As a kind of physical vapor deposition technology, magnetron sputtering technology can be used to prepare thin metal films. It gives a better control of the film thickness and has strong adhesion. Paper-based materials provide a kind of porous microstructure leading to capillary action. This is beneficial for integrating the microchannel. On the basis of keeping the original space structure, if a metal film with appropriate thickness can be constructed over the paper, an electrode with three-dimensional structure will be developed. This is useful in helping the working electrode to accumulate the target ions and then improve the detection efficiency. Based on these characteristics, in this paper, nitrocellulose membrane (NC) with regular dimensional structure was used as the substrate, and magnetron sputtering technology was applied to fabricate the sensor chip for high-sensitivity and low-cost heavy metal ion detection by the electrochemical method.

## 2. Materials and Methods

### 2.1. Chemicals and Reagents

NC paper (0.45 μm) was purchased from GE Healthcare (Freiburg, Germany). Copper(II), lead(II), cadmium(II), zinc(II), and bismuth(III) solutions were purchased from Institute for Environment Reference Materials Ministry of Environmental Protection (Beijing, China); acetic acid (HAC), anhydrous sodium acetate (NaAC), potassium chloride (KCl), potassium ferricyanide (K_3_[Fe(CN)_6_]), and potassium ferrocyanide (K_4_[Fe(CN)_6_]) were obtained from Xi Long Chemical Cn. (Guangdong, China); and 95% concentrated sulfuric acid (H_2_SO_4_) was obtained from Beijing Chemical Works (Beijing, China). All chemical reagents were of analytical grade, and the water used in the experiments was deionized water. All the experiments were done at room temperature.

### 2.2. Apparatus

Magnetron sputtering equipment was purchased from SKY Technology Development (Shenyang, China). Characterization of the electrodes’ surface by scanning electron microscopy (SEM) and component analysis by energy-dispersive X-ray spectroscopy (EDS) were performed using an S-4800 emission microscope (Tokyo, Japan). A Gamry Reference 600 electrochemical analyzer was used for the electrochemistry analysis (Warminster, PA, USA). A digital pH meter (PHS-3C) was acquired from Shanghai Instrument Electric Science Instrument Limited by Share Ltd. (Shanghai, China). Water quality workstations (Direct-Q3) were purchased from Millipore Company (Darmstadt, Germany).

### 2.3. Fabrication of Paper-Based Electrodes

**Figure 1:**
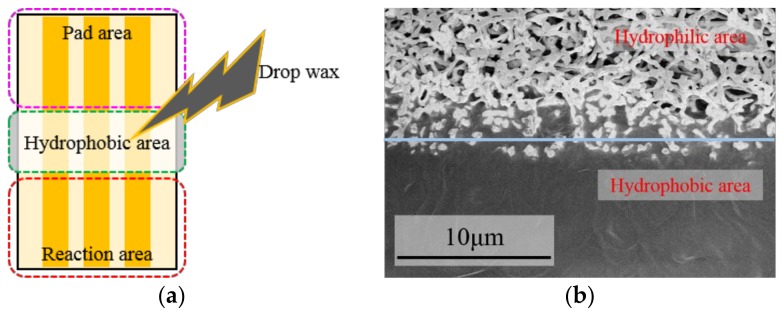
(**a**) Image of paper-based electrode chip and (**b**) SEM image of the electrode top surface of the junction of the hydrophilic/hydrophobic areas after hydrophobic treatment.

NC paper has a uniform and porous structure with apertures of 0.45 μm. Firstly, the mask plate within 1 mm thickness was designed and prepared by mechanical processing using stainless steel or 3D printing using UV-curable resin (SOMOS8000). Secondly, the paper-based sensor electrode was fabricated using magnetron sputtering technology. The thickness of the sputtered Au was 750 Å. After that, an Ag/AgCl slurry was coated on the reference electrode, and then thermal cured at 80 °C for 10 min to form a simple disposable RE. At last, hydrophobic treatment with wax was performed to isolate the reaction area and pad area of the electrode chip due to the hydrophilic nature of NC paper, so that the electrochemical reaction area was defined. Wax was heated to melting and then dropped on the hydrophobic area. When the wax was solidified, effective hydrophilic/hydrophobic areas were formed on the NC paper. As shown in Figure 1a, the paper-based electrode chip was divided into pad area, hydrophobic area, and reaction area after hydrophobic treatment. Figure 1b shows the top surface of the junction of the hydrophilic/hydrophobic areas.

### 2.4. Package of the Electrode System

**Figure 2:**
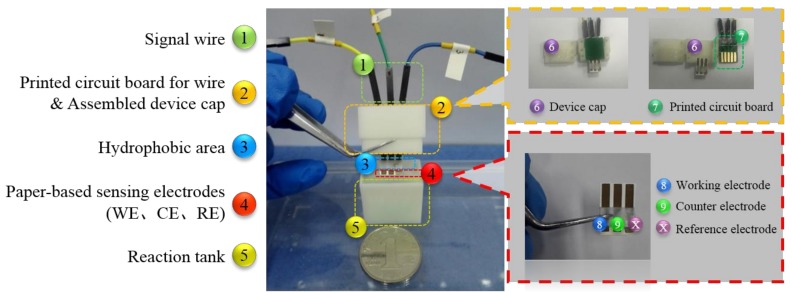
Images of paper-based sensor chip and the portable detection device.

NC paper is a kind of flexible material, and the signal wire is difficult to connect using ultrasonic bonding or thermo-compression bonding technology. In this paper, a printed circuit board was designed. It can contact closely with the paper-based electrodes, so applying the driving voltage and acquiring the detection signal can be realized. The portable detection device was produced from UV-curable resin (SOMOS8000) with low-viscosity material through 3D printing. The whole experimental device is shown in Figure 2.

### 2.5. Square-Wave Stripping Voltammetry Measurement of Cu^2+^

Cu^2+^ sample solutions were prepared using HAC–NaAC buffer solution. The concentration of HAC–NaAC in the Cu^2+^ sample solution was 0.1 mol·L^−1^. 

The concentration of Cu^2+^ was determined by square-wave stripping voltammetry (SWSV). There were three steps, and the parameters were set to (1) cleaning: potential 1 V, time 60 s; (2) accumulation: potential −0.2 V, time 120 s; (3) detection by square wave plus voltammetry: range −0.2–+0.8 V, frequency 25 Hz, pulse size 25 mV, step size 5 mV.

## 3. Results and Discussion

### 3.1. Characterization of Paper-Based Electrodes

#### 3.1.1. SEM and EDS Characterization

**Figure 3:**
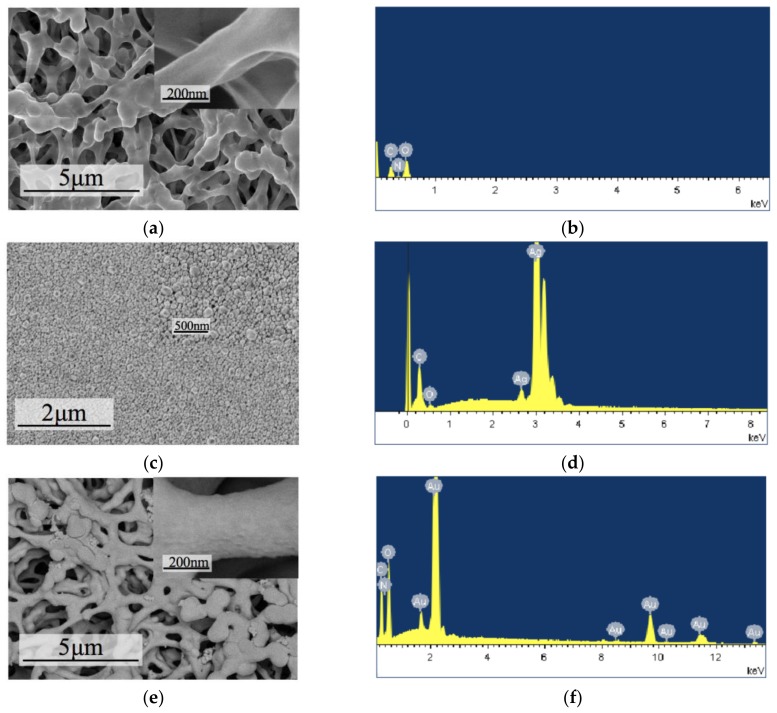
SEM and EDS images of (**a**,**b**) nitrocellulose membrane (NC) paper, (**c**,**d**) inkjet-printed silver electrode, and (**e**,**f**) magnetron-sputtered Au electrode.

The paper-based sensor electrodes were characterized by scanning electron microscopy (SEM) and energy-dispersive X-ray spectroscopy (EDS). The results are shown in Figure 3.

The NC paper had a regular three-dimensional porous network structure in microscopic vision, and the fiber surface was smooth (due to the insulation of the NC paper, it was pretreated by Pt before SEM detection).

Paper-based electrodes prepared by inkjet printing had good conductivity, but the silver particles were dense and uniformly distributed on the surface. There was no porous structure which was analogous with rigid-based materials.

After sputtering with Au, the spatial structure was similar to that of the NC paper, but there were Au particles adhered to the surface, which looked like a 3D-structured electrode.

#### 3.1.2. Electrochemical Characteristics

Three electrodes—WE, CE, and RE—were integrated on this paper-based sensor chip. Their respective electrochemical performances are discussed in the following. Three electrodes were tested in each experiment, and each experiment was performed three times.

(1) Working Electrode

**Figure 4:**
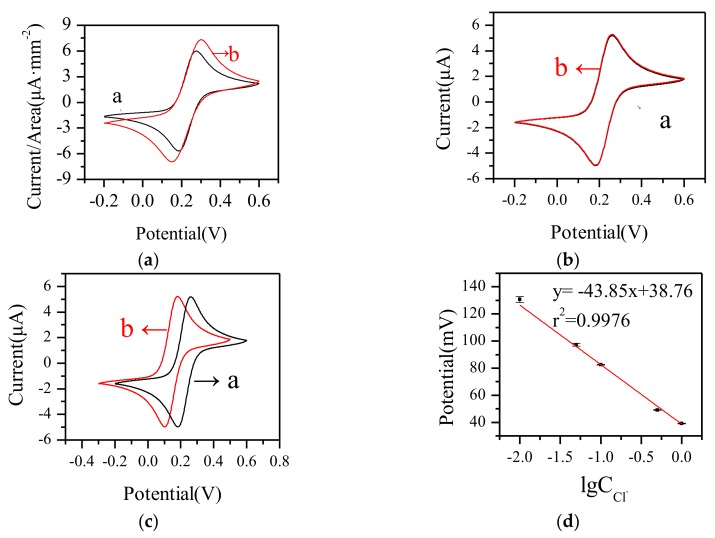
CV curves of (**a**) WE, (**b**) CE, and (**c**) RE using commercial electrodes (black curves) and paper-based electrodes (red curves) in 2.5 mM Fe(CN)_6_^3−/4−^ solution at 50 mV·s^−1^ rate and (**d**) the Nernst Response Diagram of the paper-based RE in different concentrations of Cl^−^.

The electrochemical characteristics of the paper-based Au WE were investigated and compared with a commercial Au WE by cyclic voltammetry (CV) in 2.5 mM Fe(CN)_6_^3−/4−^ solution. A commercial Pt electrode and an Ag/AgCl electrode were used as the counter electrode and reference electrode. As shown in Figure 4a, the paper-based electrode had larger response current density, and the relative standard deviation (RSD) of the paper-based WE was 5.21% (*n* = 3) in 2.5 mM Fe(CN)_6_^3−/4−^ solution. As the porous structure increased the effective surface area of the working electrode, it was useful to the electrochemical reaction, which helped to increase the effective working area and enhance the response current.

(2) Counter Electrode

The main function of the counter electrode is to conduct current. The electrochemical characterization of the paper-based Au electrode using as a counter electrode was investigated by cyclic voltammetry and compared with the commercial Pt electrode. A commercial Au electrode and an Ag/AgCl electrode were used as the working electrode and reference electrode. The RSD of the paper-based CE was 1.48% (*n* = 3) in 2.5 mM Fe(CN)_6_^3−/4−^ solution. As shown in Figure 4b, the cyclic voltammetric curves of the paper-based Au counter electrode and commercial Pt electrode were similar. This indicated that the paper-based Au electrode had good conductivity and was effective in current conduction as a CE.

(3) Reference Electrode

The feasibility of the simple reference electrode was verified as shown in Figure 4c. The paper-based RE and an Ag/AgCl electrode were used as reference electrodes with a commercial Au working electrode and a Pt counter electrode. The redox peak current value of the paper-based RE was consistent with that of the Ag/AgCl RE., while the redox peak position was negatively shifted in the negative direction. This might be because of the concentration of Cl^−^. As shown in Figure 4d, the bigger the concentration of Cl^−^ was, the less the drift was. 

**Figure 5:**
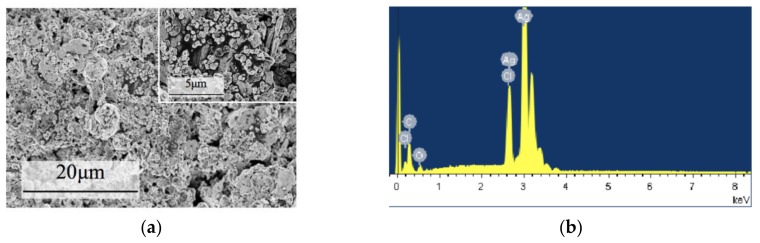
(**a**) SEM and (**b**) EDS of the paper-based RE.

Figure 5a shows the surface morphology of the paper-based RE. This RE mostly contained Ag and Cl elements (Figure 5b), which promoted the properties of the reference electrode.

**Figure 6:**
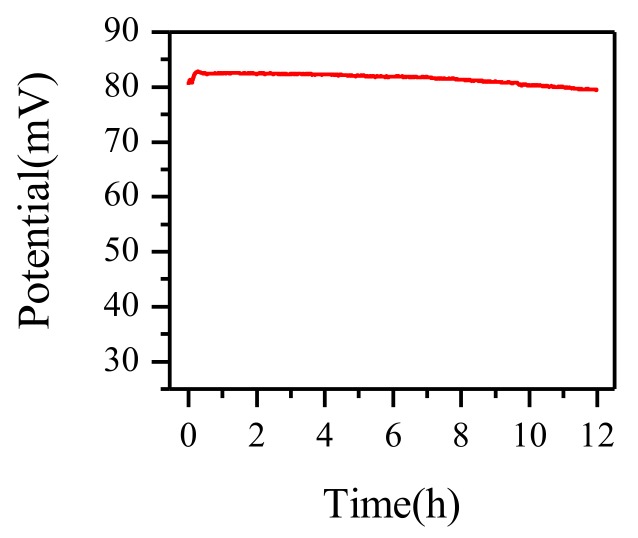
Stability of the paper-based RE in 0.1 M KCl solution over 12 h.

Potential stability was an important parameter in characterizing the RE. A well-operated RE must have little potential drift over time. As shown in Figure 6, during 12 h, the max drift value of the paper-based RE was 3.2 mV. The average of the per hour drift was only 0.27 mV. As for the reproducibility, the RSD of the paper-based RE was 0.37% (*n* = 3) in 2.5 mM Fe(CN)_6_^3−/4−^ solution.

Above all, the paper-based RE can be considered to have good stability and to be a suitable alternative to the commercial Ag/AgCl RE.

### 3.2. Optimization of Experimental Conditions

The experimental conditions were optimized in order to attain highly sensitive Cu^2+^ detection. In this paper, the effects of accumulation potential, accumulation time, and pH value on the peak current are discussed. All experiments had a single variable and the concentration of Cu^2+^ was 100 μg·L^−1^.

#### 3.2.1. Optimization of Accumulation Potential

**Figure 7:**
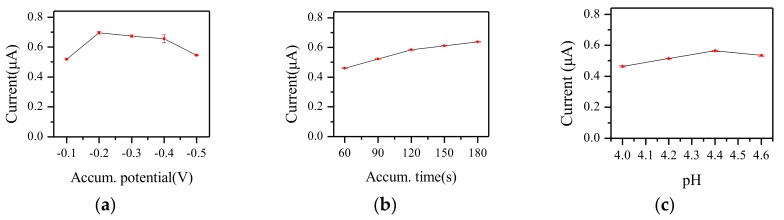
The effects of (**a**) accumulation potential, (**b**) accumulation time, and (**c**) pH value on the peak current by square-wave stripping voltammetry (SWSV) at 100 μg·L−^1^ Cu^2+^, using the paper-based sensor chip.

As shown in Figure 7a, with the accumulation potential shifting from −0.1 V to −0.2 V, the current response was increased. At −0.2 V, the response current achieved the biggest value. With the accumulation potential negative shifting further, the current response gradually decreased. The reason for this might be that the background current was also enhanced and the deposition of heavy metal ions was hindered by a hydrogen evolution reaction. Therefore, −0.2 V was selected in this work.

#### 3.2.2. Optimization of Accumulation Time

The accumulation time was changed from 60 s to 180 s. As shown in Figure 7b, the longer the accumulation time was, the bigger the response current value. However, when the accumulation time was greater than 120 s, the growth rate slowed down. This might be due to the ions enriched on the electrode surface becoming saturated. Considering the efficiency, 120 s was selected for further work.

#### 3.2.3. Optimization of pH Value

The pH value of the solution can be changed by adding HAC or NaOH, and during this process, the concentration of Cu^2+^ remained constant. As shown in Figure 7c, when the pH value was 4.4, the response current value reached the maximum. Analyzing the results, lower pH meant that the concentration of H^+^ was higher. H^+^ can be easily reduced to H_2_, and the reduction of heavy metal ions was blocked. While the pH was higher, heavy metal ions were prone to being hydrolyzed, affecting the determination. Thus, pH = 4.4 buffer solution was selected in this work.

### 3.3. Electrochemical Response to Cu^2+^ by Paper-Based Sensor Chip

Cu^2+^ was taken as the sample heavy metal ion in order to test the electrochemical properties of the paper-based sensor chip. The concentration of Cu^2+^ was tested in the range of 5–1000 μg·L^−1^. Each sample was tested three times.

**Figure 8:**
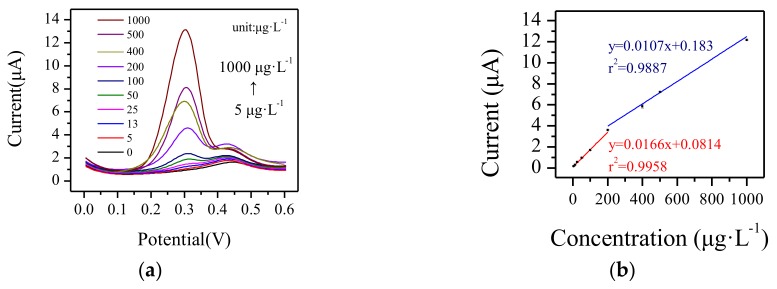
(**a**) Current response of the paper-based sensor chip in different concentrations of Cu^2+^ by SWSV, and (**b**) corresponding linear calibration plots of the stripping peak current for Cu^2+^.

As shown in Figure 8a, with the increase of the Cu^2+^ concentration, the peak current at about 0.3 V increased linearly. This paper-based sensor chip displayed two linear ranges for Cu^2+^ determination. In the range from 5 μg·L^−1^ to 200 μg·L^−1^, the sensitivity was 0.0166 μA·μg^−1^·L with a linear coefficient of 99.58%. In the range of 200–1000 μg·L^−1^, the sensitivity was 0.0107 μA·μg^−1^·L and the linear coefficient was 98.87%. The detection limit was 2 μg·L^−1^. The detection range satisfied the environmental quality standard for surface water (GB 3838-2002) in China, for which the detection range for Cu^2+^ is from 10 μg·L^−1^ to 1000 μg·L^−1^.

### 3.4. Interference Studies

**Figure 9:**
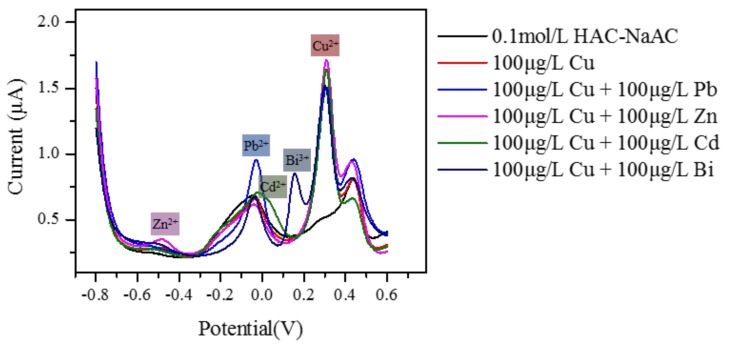
The effects of different interference ions on the response current of Cu^2+^. (In this figure, Cu means Cu^2+^, Pb means Pb^2+^, Zn means Zn^2+^, Cd means Cd^2+^, and Bi means Bi^3+^).

Pb^2+^, Cd^2+^, Zn^2+^, Bi^3+^, Cl^−^, SO_4_^2−^, CO_3_^2−^, PO_4_^3−^, Na^+^, and K^+^ were used as the interference ions. They were added to a 100 μg·L^−1^; Cu^2+^ sample solution separately. The results are shown in Figure 9 and Table 1. 

It was found that different metal ions had different redox potentials and were evidently distinguished. From Table 1, when Pb^2+^ was added dropwise to the sample solution, the response current value was reduced by about 15%. This was possibly because of the stronger adhesion of Pb^2+^ to the electrode’s surface, which weakened the response of Cu^2+^. According to Table 1, the deviation between Cu^2+^ and Cd^2+^, Zn^2+^, and other common ions was less than 10%. In addition, Bi^3+^ can be used as a sensitive membrane to increase the current value [1]. However, the peak current was affected by concentration. Above all, it can be considered that this paper-based sensing chip showed good anti-interference properties.

### 3.5. Recovery Measurement in Real Samples (Cu^2+^)

The practical application of the paper-based sensor chip was investigated using the spike recovery method. Two water samples were taken from Lake A and Lake B in Beijing, China. Standard Cu^2+^ solution was added to the water samples in different concentrations. Then, the Cu^2+^ sample solutions were mixed with HAC–NaAC buffer solution with high concentration in a ratio of 1:30 (0.25 mL:7.5 mL) to make the pH equal to 4.4 and the final concentration of HAC–NaAC was 0.1 mol·L^−1^ after mixing. The concentration of each sample was measured 3 times using SWSV. The results are shown in Table 2. The recoveries for Cu^2+^ ranged from 96% to 109%, which indicated that the paper-based sensor chip can be used for metal ion detection in real water samples.

### 3.6. Reproducibility Study

In this paper, each experiment was tested three times and the reproducibility was estimated with three different paper-based sensor chips that were prepared independently using the same method. The values of relative standard deviation (RSD) were 2.14%, 1.64%, 3.16% for 100 μg·L^−1^, 300 μg·L^−1^, and 400 μg·L^−1^ Cu^2+^ and 1.42% for 3 μg·L^−1^ Hg^2+^, respectively. All the RSD were less than 5%, which verified that the reproducibility of the paper-based chip was acceptable.

### 3.7. Other Metal Ions Detection

**Figure 10:**
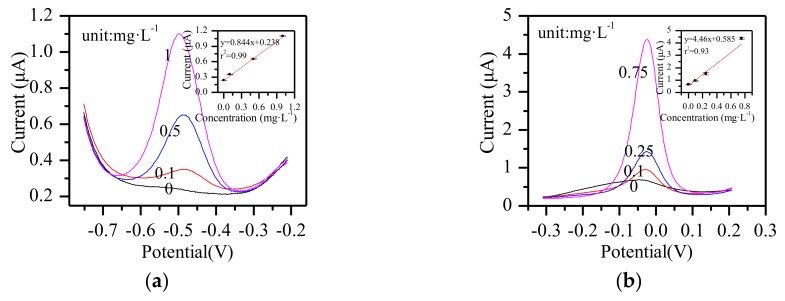

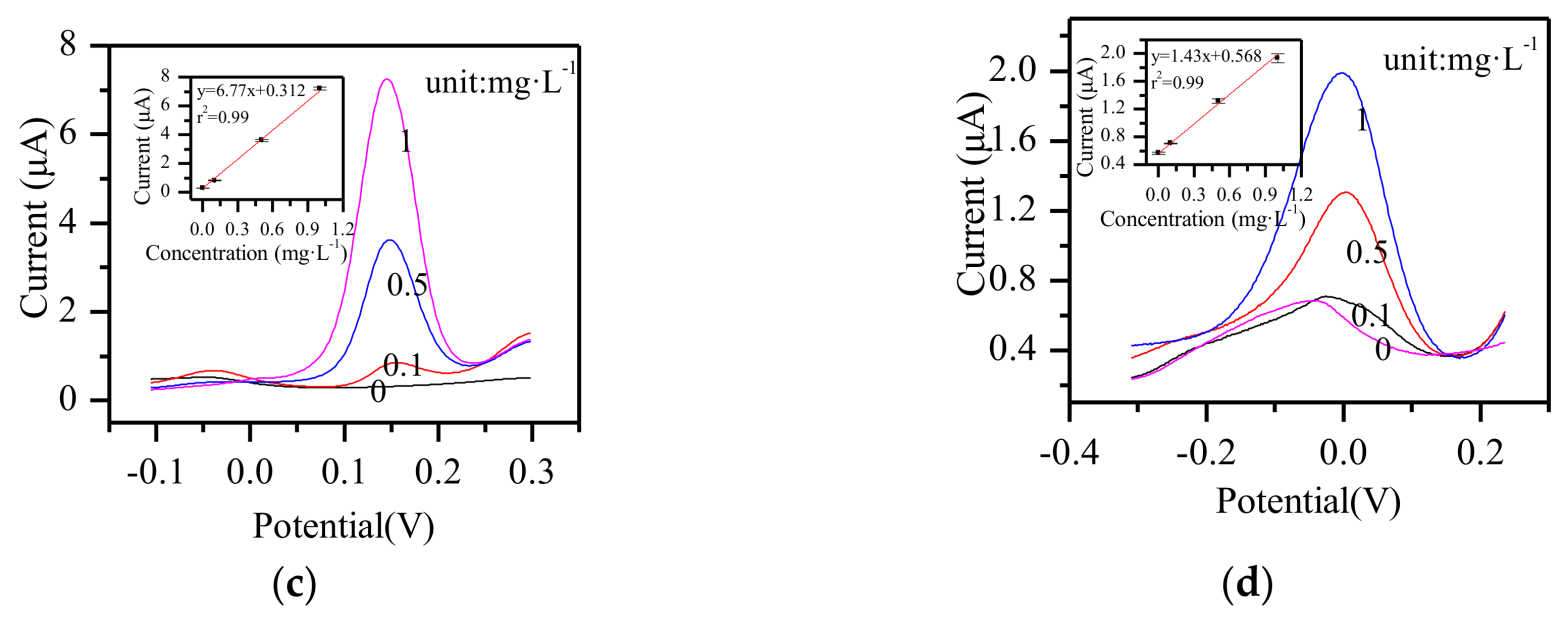
SWSV response of the paper-based sensor chip for (**a**) Zn^2+^, (**b**) Pb^2+^, (**c**) Bi^3+^, and (**d**) Cd^2+^.

Other metal ions—Zn^2+^, Pb^2+^, Bi^3+^, and Cd^2+^—were detected by the paper-based sensor chip using SWSV, and the results are shown in Figure 10. From Figure 10a, with the increase of Zn^2+^ concentration in the range 0–1 mg·L^−1^, the peak current at about −0.5 V increased linearly. The sensitivity was 0.844 μA·mg^−1^·L with a linear coefficient of 99%. As shown in Figure 10b, in the range of 0–0.75 mg·L^−1^, the peak current of Pb^2+^ at about −0.12 V increased linearly. The sensitivity was 4.46 μA·mg^−1^·L and the linear coefficient was 93%. Figure 10c showed that the peak current of Bi^3+^ grew linearly from 0 mg·L^−1^ to 1 mg·L^−1^, and the sensitivity and linear coefficient were 6.77 μA·mg^−1^·L and 99%. Figure 10d showed that the peak current of Cd^2+^ grew linearly from 0 mg·L^−1^ to 1 mg·L^−1^, and the sensitivity and linear coefficient were 1.43 μA·mg^−1^·L and 99%. In this paper, there were only four data points listed for each ion, since it was a preliminary test. In order to obtain more accurate results, more experiments should be prepared. The experimental conditions also need to be optimized separately for each different heavy metal ion. The limit of detection of each metal ion can be obtained after precise detection by the paper-based sensor chip.

## 4. Conclusions

In summary, compared with a solid electrode, this paper-based sensor chip fabricated using magnetron sputtering technology effectively took advantage of the three-dimensional porous structure of the paper, which increased the effective working area of the electrode. The experimental results indicated that the chip had high sensitivity and a wide measurement range. The detection range met the requirements of the environmental quality standard for surface water in China, for which the range for Cu^2+^ is from 10 μg·L^−1^ to 1000 μg·L^−1^. This study promotes the development of on-site rapid detection technology for heavy metal ions.

## Figures and Tables

**Table 1:** Effect of interference ions on the peak current of Cu^2+^ (100 μg·L^−1^).

Interference Ions	Concentration of Interference Ions (μg·L^−1^)	Percentage of Peak Current Compared with 100 μg·L^−1^ Cu^2+^ (%)
Pb^2+^	100	86.17 ± 6.15
	250	84.23 ± 7.44
Cd^2+^	100	103.46 ± 9.97
	500	99.59 ± 7.38
Zn^2+^	100	107.62 ± 5.11
	500	104.16 ± 6.65
Bi^3+^	100	142.95 ± 5.23
	500	102.44 ± 6.02
Cl^−^, SO_4_^2−^, CO_3_^2−^, PO_4_^3−^, Na^+^, K^+^	10^5^, 10^5^, 5 × 10^4^, 5 × 10^4^, 10^5^, 1.1 × 10^5^	92.42 ± 4.19

**Table 2:** Recovery measurement of Cu^2+^ in real samples using the paper-based sensing chip (*n* = 3).

Samples	Concentration of Added Cu^2+^ (μg·L^−1^)	Concentration Found (μg·L^−1^)	Recovery (%)
1	0	N	-
	20	21.52 ± 0.60	107.59 ± 2.99
	100	108.96 ± 1.16	108.96 ± 1.16
2	0	N	-
	40	43.42 ± 0.30	108.56% ± 0.74
	75	72.11 ± 1.13	96.14% ± 1.50

(N: not found).
